# Shock

**Published:** 1899-12-02

**Authors:** 


### SHOCK.

MR. MOYNIHAN,[1] Assistant Surgeon to the Leeds General Infirmary, says that shock is one of the chief difficulties encountered by the surgeon, and that it cannot always be foreseen to what extent it will develop in any protracted operation, and further, that when once developed the case from that moment becomes a serious one. In all cases then which even remotely threaten to exhibit indications of shock, he would, with the object of preventing the condition, use remedies which are commonly employed to overcome it after it has definitely asserted itself. Strychnine is administered hypodermically before the anæsthetic or the operation is commenced. He begins with a dose of 10 minims of the liquor strychninæ. During the operation 5 minims may be given as required. He has given as much as 30

minims, and frequently as much as 20 to 25 minims, altogether, and has never in one single instance seen any evil result, or any physiological manifestation as a sequel to such liberal administration. He also gives from 10 to 20 ounces of hot saline solution by the rectum, with or without a small quantity of brandy. As soon as the patient is under the anæsthetic the transfusion, or rather the infusion, of saline solution to the extent of 1 to 4 pints may be begun. He does not necessarily carry out in each case all the measures he suggests. His point is that he would anticipate shock, and that in so doing he would use larger doses of strychnine than are usually recommended. Mr. Basil Hall,[1] Surgeon to the Bradford Union Infirmary, writing on the treatment of shock, also recommends large doses of strychnine, saying that he has given injections of liquor strychninæ in doses varying from 15 to 20 minims on 14 occasions, and that he has never regretted the largeness of the dose. He also notes the absence of any signs of poisoning afterwards.

[1] Brit. Med. Jour., Nov. 25.

